# An empirical study on the effect of outdoor illumination and exercise intervention on Children’s vision

**DOI:** 10.3389/fpubh.2023.1270826

**Published:** 2023-12-12

**Authors:** Shuaixiong Liao, Xueying Li, Nan Bai, Danping Wu, Wenliang Yang, Feng Wang, Hao Zong Ji

**Affiliations:** ^1^Inner Mongolia University of Science and Technology, Baotou, China; ^2^Caotang Primary School, Chengdu, China; ^3^Chongqing Liangjiang Yucai Middle School, Chongqing, China

**Keywords:** exercise, retina, children, myopia, outdoor lighting, school

## Abstract

**Objective:**

To explore the relationship between outdoor lighting and sports and the development of myopia, and to analyze the effects of outdoor lighting and exercise on the diopter of children with normal vision and myopia, so as to provide guidance for the prevention and treatment of myopia in children and adolescents in the future.

**Methods:**

A total of 201 children were divided into two groups according to myopia or not. Each group was randomly divided into 4 groups: outdoor exercise group, outdoor control group, indoor exercise group and indoor control group. Among them, the outdoor exercise group and indoor exercise group received moderate and high intensity aerobic exercise 3 times a week for 60 min each time for 12 months, while the outdoor control group and indoor control group had normal study and life during the corresponding period of time. No additional exercise intervention. At the end of the experiment, the diopter of each group was compared.

**Results:**

The diopter of all groups with normal vision and myopia decreased significantly after the experiment (*p* < 0.01). There were significant differences in diopter between outdoor exercise group and indoor control group (*p* < 0.01), between outdoor exercise group and indoor control group (*p* < 0.05), and between indoor exercise group and indoor control group (*p* < 0.01). There were significant differences in diopter between indoor exercise group and indoor control group (*p* < 0.01). The differences among myopic children after the experiment showed that there was significant difference in diopter between outdoor exercise group and indoor exercise group (*p* < 0.05), between outdoor exercise group and indoor control group (*p* < 0.01), and between outdoor control group and indoor control group (*p* < 0.05). There were significant differences in the changes of diopter between the outdoor control group and the indoor exercise group with normal vision and myopia before and after the experiment (*p* < 0.05).

**Conclusion:**

Outdoor light and exercise intervention can have a beneficial effect on children’s vision, but because of whether children are myopic or not, the effect is different, outdoor light and exercise have a better effect on reducing the diopter of children with normal vision.

## 1 Introduction

Myopia is one of the most common eye diseases among children and adolescents. in 2020, the overall myopia rate of Chinese children and adolescents was 52.7%, an increase of 2.5 percentage points over 2019, including 14.3% for 6-year-old children, 35.6% for primary school students, 71.1% for junior high school students and 80.5% for high school students. Projections by Holden et al. on the global prevalence of myopia show that by 2050, there will be 4.758 billion people with myopia (49.8% of the world population) and 938 million people with high myopia (9.8% of the world population) (1). The increasing incidence of myopia year by year will further increase the risk of myopic complications such as retinal detachment, cataract, glaucoma and blindness, which will have a great impact on national security, socio-economic production activities and personal health. According to the National Visual Health report, as far as China is concerned, the socio-economic cost caused by various visual defects in 2012 alone reached more than 560 billion yuan (2). To sum up, the myopia of children and adolescents has become a major public health problem at home and abroad, so it is urgent to explore effective means and methods to prevent and cure adolescent myopia.

In recent years, researchers have made many attempts on how to effectively control the myopia of children and adolescents. Existing studies have shown that corneal plastic lens (3), surgical treatment (4) and drug therapy (5) all play a certain role in the prevention, relief and treatment of myopia, but there are also some drawbacks. For example, atropine in drug therapy can make the body develop drug resistance, and it is easy to rebound after drug withdrawal; long-term wearing of keratoplasty lens will induce keratitis; surgical treatment will also face the risk of blindness after failure. Therefore, how to solve the problem of myopia safely and effectively is still a clinical bottleneck that needs to be broken through. The above troubles force us to explore more safe and effective means of prevention and treatment of myopia in children and adolescents. With the deepening of the study of the relationship between exercise and health, the role of exercise in vision protection has gradually become the focus of academic attention (6). Previous studies have shown that exercise can relieve eye fatigue by promoting human metabolism and accelerating eye blood circulation, and it can also enhance the contractile strength and adjustment ability of intraocular regulatory muscles and extraocular convergent muscles. Make the regulation and axis of the eye more coordinated (7–9). Based on the above findings, more and more scholars try to reduce the rate of myopia in children and adolescents through exercise.

In addition to exercise, studies have found that outdoor light can also have a good effect on vision. It is reported that outdoor light can effectively prevent myopia by stimulating the release of dopamine in the retina, inhibiting the elongation of the eye axis and effectively preventing myopia (10, 11). On the other hand, it can also increase the level of vitamin D in the blood, resulting in differences in individual vitamin D levels, thus achieving the regulation of myopia (12). Both exercise and outdoor lighting have beneficial effects on myopia, but who plays a key role has always been controversial. At present, the latest foreign reports show that compared with exercise, outdoor lighting may be the most important factor in the prevention and treatment of myopia, but the relevant conclusions still need to be further confirmed by research (13).

To sum up, the situation of myopia among students is becoming more and more serious, how to effectively prevent and cure myopia has become an urgent problem to be solved. At present, there is a dispute about the prevention and treatment mechanism of myopia caused by outdoor sports, and the focus of the debate is whether exercise itself or outdoor light plays a major role in the prevention and treatment of myopia. Therefore, whether simple exercise or outdoor lighting or the superposition of outdoor lighting and exercise is the key to the prevention and treatment of myopia, which needs further study. In addition, for different groups of people, such as myopia or normal vision, there are few studies on whether outdoor light and exercise have different effects on the prevention and treatment of myopia. Based on this, this subject intends to take primary school students in Chengdu as the research object to explore the correlation between sports and outdoor lighting and the occurrence and development of myopia, and to further explore the effects of outdoor and sports on people with different eyesight. to provide reference for the prevention and treatment of myopia in children and adolescents.

## 2 Methods

### 2.1 Study design and participants

**Figure 1:**
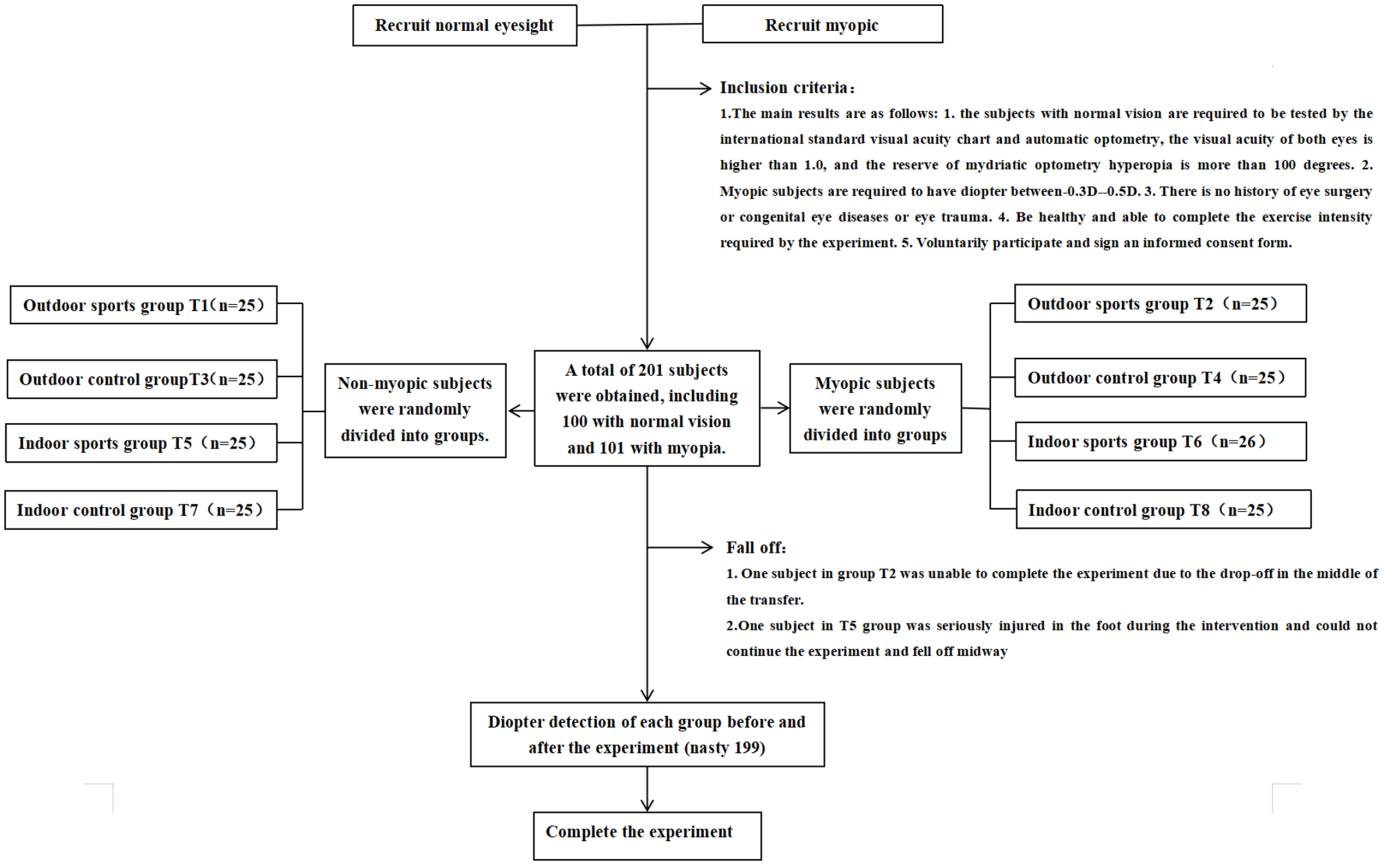
Experimental flow.

We conducted a randomized controlled trial on the effects of outdoor light and exercise intervention on children’s vision in a primary school in Chengdu from March 2022 to March 2023. This research procedure is in line with the principles of the Helsinki Declaration, and has been approved by the Medical Ethics Committee of Chongqing Southwest University Hospital (approval number: 202215), and the study has been completed in accordance with the ethical requirements of clinical trials. an informed consent form was signed with each subject and his guardian before the start of the experiment. According to the pre-established inclusion criteria, 201 students were recruited, including 100 students with normal vision and 101 students with mild myopia (−3.0D < binocular diopter < −0.5D). The students with normal vision and myopia were randomly divided into four groups: outdoor exercise group (T1 group), outdoor control group (T3 group), indoor exercise group (T5 group) and indoor control group (T7 group). Myopic subjects were randomly divided into outdoor exercise group (T2 group), outdoor control group (T4 group), indoor exercise group (T6 group) and indoor control group (T8 group). The specific grouping process is shown in Figure 1. The random packet sequence is generated by the random number generation algorithm on the computer to ensure the scientific distribution.

### 2.2 Intervention

The purpose of this study is to make use of the students’ delayed service time after class to carry out the experiment, which requires students to carry out corresponding sports and outdoor light exposure according to the prior grouping arrangement. In the outdoor exercise group (T1, T2), the participants were required to do moderate and high intensity aerobic exercise in the outdoor playground for 12 weeks, three times a week for 60 min each time, while in the outdoor control group (T3, T4), the participants were asked to stay outdoors during the experiment without exercise intervention. In the indoor exercise group (T5, T6), participants were required to do moderate and high intensity aerobic exercise indoors 3 times a week for 60 min each time for 12 weeks. In the indoor control group (T7, T8), the participants were asked to stay indoors during the experiment without exercise intervention. The specific intervention methods of each group are shown in Table 1. The subjects and data collectors were not informed of the purpose of this study throughout the experiment.

**Table 1:** Experimental intervention in different groups.

Group		Experimental intervention
Outdoor exercise group (T1、T2)	Frequency Intensity Content	3 times a week, 60 min each time, a total of 12 months. HRmax60% ~ 70% moderate intensity aerobic exercise was performed in the adaptation stage (1–2 months), High-intensity aerobic exercise in HRmax80% ~ 85% during the experimental stage (2–12 months). Exercise in the outdoor playground, the specific sports intervention is as follows: Monday is mainly endurance training, training content includes 4 × 25 m back and forth running, 400 m running, rope skipping, each project continues 15 min, inter-group rest 2 min. Wednesday is mainly strength training, including sit-ups, frog jump, bow and arrow steps, kneeling push-ups, head-hugging squats and so on. Each project continues to 8 min, and there is a 1 min break between groups. Friday is mainly agile and coordinated training, including rope ladder training, obstacle running, balance beam walking, small hurdle training, etc., each project continues 8 min, inter-group rest 1 min
Outdoor control group (T3、T4)	Frequency Intensity Content	3 times a week, 60 min each time, a total of 12 months. Carry out very low intensity (exercise intensity < HRmax50%) free leisure activities outdoors Take a walk, play chess, chat, etc
Indoor exercise group (T5、T6)	Frequency Intensity Content	3 times a week, 60 min each time, a total of 12 months. HRmax60% ~ 70% moderate intensity aerobic exercise was performed in the adaptation stage (1–2 months), High-intensity aerobic exercise in HRmax80% ~ 85% during the experimental stage (2–12 months). Exercise in the indoor training hall, the specific sports intervention is as follows: Monday is mainly endurance training, training content includes 4 × 25 m round-trip running, 400 m running, rope skipping, each project continues 15 min, inter-group rest 2 min. Wednesday is mainly strength training, including sit-ups, frog jump, bow and arrow steps, kneeling push-ups, head-hugging squats and so on. Each project continues to 8 min, and there is a 1 min break between groups. Friday is mainly agile and coordinated training, including rope ladder training, obstacle running, balance beam walking, small hurdle training, etc., each project continues 8 min, inter-group rest 1 min
Indoor control group (T7、T8)	Frequency Intensity Content	3 times a week, 60 min each time, a total of 12 months. Carry out very low intensity (exercise intensity < HRmax50%) free leisure activities indoors Listen to music, play chess, chat, etc.

### 2.3 Data collection

In this study, two full-time ophthalmologists used the method of mydriatic refractive test to detect the diopter of the subjects before and after the experiment, specifically choosing compound tropicamide eye drops as a drug for ciliary paralysis, once per 5 min for a total of 3 times. After 20 min, doctors judged the condition of ciliary paralysis according to the pupil light reflex, with no pupil light reflex and pupil diameter greater than 6 mm as the judgment standard. Half an hour later, the diopter (SEQ) was examined by a full-time ophthalmologist. Each subject was tested for 3 times in the left and right eyes, and the average value of the effective measurement was taken for 3 times. The whole testing process is carried out in the hospital, and the measured data are collected by doctors.

### 2.4 Statistical analysis

SPSS25.0 was used to analyze the experimental data, variance homogeneity test was used to screen the data, and the average ± standard deviation (M ± SD) was used to express the continuous variables in accordance with normal distribution, and the data were accurate to two decimal places. The data processing of each group before and after the experiment, before and after the experiment, using paired sample T test; comparison between groups, using single-factor ANOVA analysis of variance, the process of normal distribution and variance homogeneity Bartlett test, and then single-factor analysis of variance, through LSD or Dunnett T3 for post-test, and using Bonferroni to correct *p* value. Independent sample T test was used to compare the difference of dioptre between normal vision and myopic subjects before and after the experiment. In the process, the normal distribution and homogeneity of variance of the data were tested, and then LSD or Dunnett T3 was used for post-test. Finally, Bonferroni was used to correct the *p* value. *p* < 0.05 means there is significant difference, *p* < 0.01 means that there is a very significant difference.

## 3 Results

### 3.1 Baseline characteristics of experimental subjects

As shown in Table 2, there were no significant differences in age (*p* > 0.05), sex composition (*p* > 0.05) and dioptre (*p* > 0.05) among T1, T3, T5, and T7 groups with normal visual acuity before intervention. There were no significant differences in age (*p* > 0.05), sex composition (*p* > 0.05) and dioptre (*p* > 0.05) among myopic subjects, that is, T2, T4, T6, and T8 groups (Table 2).

**Table 2:** Baseline of subjects before experiment.

	Normal eyesight				Myopia				*P*_1_/*P*_2_
	T1	T3	T5	T7	T2	T4	T6	T8	
Age (Years)	6.40 ± 0.50	6.44 ± 0.51	6.46 ± 0.51	6.48 ± 0.51	6.50 ± 0.50	6.56 ± 0.51	6.46 ± 0.50	6.48 ± 0.50	0.95/0.91
Gender (M/W)	13/12	13/12	14/10	13/12	11/13	14/11	13/13	16/9	0.96/0.60
Dioptre (D)	1.56 ± 0.25	1.52 ± 0.20	1.62 ± 0.23	1.49 ± 0.23	−1.44 ± 0.48	−1.39 ± 0.53	−1.56 ± 0.57	−1.60 ± 0.58	0.23/0.49

T1 and T2 represent outdoor exercise group, T3 and T4 represent outdoor control group, T5 and T6 represent indoor exercise group, T7 and T8 represent indoor control group, P1 is significant difference between normal vision groups, P2 is significant difference between myopia groups, *means *p* < 0.05, *means *p* < 0.01.

### 3.2 Comparison of dioptre of subjects with normal vision before and after the experiment

At the end of the experiment, the differences among different groups with normal vision showed that the dioptre after the experiment was significantly lower than that before the experiment (*p* < 0.01). The results showed that there were significant differences in dioptre between outdoor exercise group and indoor control group (*p* < 0.01), between outdoor control group and indoor control group (*p* < 0.05), and between indoor exercise group and indoor control group (*p* < 0.01). There was no significant difference in dioptre between outdoor exercise group and outdoor control group, outdoor exercise group and indoor exercise group, and between outdoor control group and indoor exercise group (*p* > 0.05) (Table 3).

**Table 3:** Changes of dioptre of subjects with normal visual acuity before and after experiment.

Group	Dioptre before experiment (D)	Post-experimental dioptre (D)	*P*_#_	*P*_&_	*P*_∮_	*P*_£_	*P*_§_
Outdoor exercise group	1.56 ± 0.25	1.26 ± 0.28	0.000**	—	0.148	0.997	0.001**
Outdoor control group	1.51 ± 0.20	1.12 ± 0.14	0.000**	—	—	0.215	0.031*
Indoor exercise group	1.64 ± 0.24	1.23 ± 0.21	0.000**	—	—	—	0.001**
Indoor control group	1.52 ± 0.22	0.95 ± 0.24	0.000**	—	—	—	—

# means comparison of differences within the group, & means comparison with outdoor sports group, ∮means comparison with outdoor control group, £ means comparison with indoor sports group, § means comparison with indoor control group, *means *p* < 0.05, **means *p* < 0.01.

### 3.3 Comparison of dioptre of myopic subjects before and after the experiment

At the end of the experiment, the differences among different groups of myopic subjects showed that the dioptre after the experiment was significantly lower than that before the experiment (*p* < 0.01). The results showed that there was significant difference in dioptre between outdoor exercise group and indoor exercise group (*p* < 0.05), between outdoor exercise group and indoor control group (*p* < 0.006), and between outdoor control group and indoor control group (*p* < 0.05). There was no significant difference in dioptre between outdoor exercise group and outdoor control group, outdoor exercise group and indoor exercise group, and between indoor exercise group and indoor control group (*p* > 0.05) (Table 4).

**Table 4:** Changes of dioptre of myopic subjects before and after experiment.

Group	Dioptre before experiment (D)	Post-experimental dioptre (D)	*P*_#_	*P*_&_	*P*_∮_	*P*_£_	*P*_§_
Outdoor exercise group	−1.44 ± 0.48	−1.79 ± 0.63	0.000**	—	0.483	0.045*	0.006**
Outdoor control group	−1.39 ± 0.53	−1.91 ± 0.54	0.000**	—	—	0.187	0.036*
Indoor exercise group	−1.57 ± 0.56	−2.13 ± 0.59	0.000**	—	—	—	0.414
Indoor control group	−1.59 ± 0.57	−2.26 ± 0.59	0.000**	—	—	—	—

# means comparison of differences within the group, & means comparison with outdoor sports group, ∮means comparison with outdoor control group, £ means comparison with indoor sports group, § means comparison with indoor control group, *means *P* < 0.05, **means *P* < 0.01.

### 3.4 Comparison of dioptre difference between normal vision and myopic subjects before and after the experiment

The study showed that after 12 months of experimental intervention, there was a significant difference in the change of dioptre before and after the experiment between the outdoor control group with normal vision and the myopic outdoor control group (*p* < 0.05). There was a significant difference in the change of dioptre before and after the experiment between the indoor exercise group with normal vision and the myopic indoor exercise group (*p* < 0.05). It is inferred that the effect of outdoor environment or sports training on myopia prevention and treatment of people with normal vision is better than that of myopia people (Table 5).

**Table 5:** Comparison of dioptre difference between normal visual acuity and myopic subjects before and after the experiment.

Group	Dioptre difference before and after experiment in normal vision group (D)	Dioptre difference before and after experiment in myopia group (D)	*F*	*P*
Outdoor exercise (normal vision vs. myopia)	−0.29 ± 0.09	−0.35 ± 0.21	11.74	0.266
Outdoor control (normal vision vs. myopia)	−0.39 ± 0.19	−0.51 ± 0.15	2.26	0.017*
Indoor exercise (normal vision vs. myopia)	−0.41 ± 0.16	−0.56 ± 0.27	6.93	0.022*
Indoor control (normal vision vs. myopia)	−0.56 ± 0.16	−0.67 ± 0.30	7.39	0.145

*means *P* < 0.05, **means *P* < 0.01.

## 4 Discussion

Myopia is a common eye disease, its development will cause irreversible damage to individual eyes, and in severe cases, it will also lead to blindness. Therefore, how to effectively prevent myopia is the focus of attention from all walks of life at this stage. Many studies have shown that outdoor sports can have a good effect on the prevention and control of myopia, such as inhibiting the growth of eye axis, promoting eye blood circulation and relieving eye fatigue, and some studies are devoted to in-depth analysis. to explore the separate benefits of outdoor and exercise for the prevention and treatment of myopia, the related research results are controversial. For this reason, this study conducted a randomized controlled trial in a primary school in Chengdu, China, to explore the effects of outdoor environment and physical exercise on the eyesight of school-age children, and to further clarify the relationship between outdoor and exercise and myopia.

Our study found that with the increase of children’s age, whether they have normal vision or myopia, their eyeball dioptre shows a downward trend. Sports and outdoor environment can not completely stop the decline of children’s dioptre, but it can effectively slow down the rate of decline and delay the occurrence of myopia in children. This is similar to the results of Guggenheim et al. (14) which pointed out in a prospective cohort study on the incidence of myopia in school-age children that physical activity and the length of time spent outdoors were significantly associated with the decline in the incidence of myopia in children. Studies have shown that the benefits of sports in the prevention and treatment of myopia are mainly attributed to the improvement of eye-related muscle function and blood circulation during exercise, the decrease of intraocular pressure and the increase of choroidal blood flow velocity after exercise, which makes the retinal blood supply sufficient. A higher level of retinal blood supply can effectively promote the development of children’s eye nerves and muscles (15, 16). As early as 2002, Mutti et al. (17) found in a 5-year cohort study that higher frequency and intensity of physical exercise is a protective factor for the eyesight of children and adolescents. Follow-up studies also confirmed that there is an intensity-time dose relationship between physical exercise and myopia prevention and control. Chinese scholar Chen Dingyan (18) found that medium-intensity physical exercise is the best in protecting the eyesight of children and adolescents. Xu Shaojun et al. (19)pointed out that only the duration of physical exercise is more than 1 h can play a role in the prevention and control of myopia. In this study, the exercise intensity was medium and high intensity (HRmax80% ~ 85%), and the exercise duration was 1 h. The results showed that exercise had a significant effect on alleviating children’s myopia. The exercise stimulation with high intensity can mobilize the blood stored under the skin or viscera to a greater extent, which increases the circulating blood volume of the body per unit time, which promotes the improvement of the blood supply of eye-related muscles to a certain extent. and then play the effect of alleviating children’s myopia. However, it should be pointed out that the improvement effect of exercise on myopia observed in our study is limited to non-myopic children, and we have not found that exercise has a significant effect on myopia relief in myopic children. This may be related to the good development of eye-related nerves and muscles that can not reverse the increased axial length of the eye. Similar to our observation in myopic children, Lundberg et al. (20) used ActiGraph accelerometer to monitor the amount and intensity of physical activity in a prospective study of the relationship between physical activity and myopia in 307 Danish children. After excluding confounding factors such as age and sex, linear regression did not find the correlation between physical exercise and the increase of children’s eyeball diopter and average axial length. Although in this experiment, Lundberg and other ActiGraph accelerometers used to measure sports have limitations, they can not effectively monitor children’s swimming, cycling and other weight-bearing exercise, which leads to the underestimation of the amount of exercise. The internal relationship between sports and myopia and the effects of sports on different eyesight groups need to be further studied.

In the study of outdoor sports and myopia, a large number of studies have pointed out that outdoor environment is the most important factor independent of sports affecting children’s myopia. A systematic study conducted by the University of Cambridge in the United Kingdom shows that children who spend an extra hour a week outdoors reduce their risk of myopia by 2%, and if they spend an extra hour a day outdoors, the risk of myopia is reduced by 13% (21). Our study also observed that no matter the subjects with normal vision or myopia, there was a significant difference in the refractive value between the outdoor control group and the indoor control group after the experiment, which suggests that outdoor factors can effectively prevent the development of myopia in children. Some studies have pointed out that the improvement of myopia in the outdoor environment may be related to the peripheral defocus of the retina induced by the environment. In the outdoor environment, the object is usually far away from the eyeball, and the refractive environment of the object tends to be consistent. Therefore, when outdoors, peripheral objects are defocused to a minimum (22). On the contrary, when indoors, the object is usually closer to the eyeball, resulting in a greater distance of refractive changes in the eyeball. In this refractive environment, the object may produce greater hyperopia defocus. It is this kind of hyperopic defocus around the retina that leads to the growth of the eye axis and the deepening of myopia in children.

Other studies have pointed out that the improvement of myopia by outdoor environment mainly lies in the lighting factors in outdoor environment, and is directly related to the duration and intensity of outdoor light. Read et al. (23) found that there was a significant difference in the prevalence of myopia between Australia and Singapore. The overall myopia prevalence rate of Australian children was significantly lower than that of Singaporean children. Further studies showed that this difference was due to the difference in daily outdoor light patterns between Australian children and Singaporean children. The former was 105 ± 6.42 min/ days, and the latter was 61 ± 6.40 min/ days. Morgan et al. (24) conducted a randomized controlled trial in Taiwan to explore the relationship between myopia prevention and outdoor light intensity. A total of 693 first-year students from 16 primary schools were enrolled in the randomized controlled trial to strictly monitor the outdoor light intensity of the students during their one-year stay in school. It was found that the change of eye axis length after 1 year was related to the intensity and duration of outdoor light. Specifically, students spending enough time outdoors under medium to high intensity light can effectively slow down the development of myopia. And compared with medium intensity light (1,000 lux-3000 lux) under high intensity light (>10,000 lux), the inhibitory effect of myopia can be obtained for a shorter time. The prevention and treatment mechanism of light on myopia is mainly attributed to the “light-dopamine” hypothesis. Dopamine, as an important neurotransmitter, can effectively inhibit the increase of eye axis length. In animal experiments, it was observed that the rate of dopamine release from chicken retina increased logarithmically with the increase of light intensity (25). Increasing outdoor light can prevent myopia by regulating the secretion of dopamine in the retina and then affecting the length of the eye axis (26).

In addition to observing the significant inhibitory effect of outdoor light and exercise on the development of myopia in school-age children, the study also found that there were differences in the reducing effect of outdoor light or exercise on the diopter of children with different vision (normal vision vs. myopia). Specifically, we compared the difference of diopter between normal vision subjects and myopic subjects who took the same intervention. The study found that compared with myopic subjects, outdoor light or exercise intervention had a better effect on reducing diopter in subjects with normal vision. Our results are similar to the results of a meta-analysis by Xiong et al. (27) which included 25 experiments related to outdoor lighting and myopia. It is concluded that outdoor factors are more effective in the prevention and treatment of myopia in non-myopic individuals. Also on the meta-analysis of the relationship between outdoor lighting and myopia, Ho et al. (28) also found that outdoor lighting factors significantly inhibit the growth of eye axis length in both myopic and non-myopic individuals, but different from our results, Ho and other studies pointed out that outdoor lighting factors are more effective in slowing down the development of myopia than non-myopic individuals. Among the 13 studies included in the analysis by Ho et al., there are some studies in which the daily outdoor time of children is evaluated by sending questionnaires to their parents. The subjective recall of parents can not accurately measure the level of outdoor light exposure of children during the study, resulting in underestimation or overestimation, resulting in the deviation of the research data, and then affect the results of meta-analysis. This may be the reason why the results of Ho and other studies are different from ours.

In short, our study objectively explored the effects of outdoor and exercise on eyeball diopter in children with non-myopia and myopia through randomized controlled trials. The study suggests that more outdoor sports before children are myopic can prevent the occurrence of myopia to the greatest extent; for myopic children, staying outdoors or in an environment with sufficient light is more effective in slowing down the further development of myopia. This study also has some limitations, the study does not use the relevant instruments to accurately monitor the environmental light level and the amount of physical activity of the subjects, and children’s exercise participation and outdoor light exposure duration can not be controlled during weekends and holidays, which may to some extent lead to the overestimation of outdoor light intensity in overcast and rainy days and the underestimation of the amount of exercise in the exercise control group. In addition, we also failed to evaluate the length of close eye use during the experiment, which may lead to overestimation of the impact of outdoor and sports factors on the growth of myopia in the indoor control group.

Future research should design more scientific and rigorous large-scale animal or human experiments, through the strict detection and regulation of outdoor light variables and movement variables to verify the applicability of the current research conclusions. In addition, whether there is the most suitable light intensity and exercise intensity to improve myopia, and under the most suitable conditions, which of the above two factors have a better effect on myopia needs to be studied and discussed. In addition, gender, race and genetic factors should also be taken into account, and the impact of the differences in these factors on the results needs to be further confirmed.

## 5 Conclusion

Outdoor light and exercise intervention lasting for 12 months can significantly inhibit the decrease of children’s eyeball dioptre and improve myopia. In terms of individual benefits, outdoor lighting factors have a significant effect on the reduction of eyeball dioptre in both myopic and non-myopic children, while exercise intervention factors are only effective in reducing dioptre in non-myopic children. Outdoor light and exercise intervention have different effects on dioptre of children with different visual acuity. Compared with myopic children, the effect of outdoor light and exercise intervention on dioptre of children with normal vision is better.

## Data availability statement

The raw data supporting the conclusions of this article will be made available by the authors, without undue reservation.

## Ethics statement

The studies involving humans were approved by the Medical Ethics Committee of Southwest University Hospital of Chongqing. The studies were conducted in accordance with the local legislation and institutional requirements. Written informed consent for participation in this study was provided by the participants’ legal guardians/next of kin. Written informed consent was obtained from the individual(s), and minor(s)’ legal guardian/next of kin, for the publication of any potentially identifiable images or data included in this article.

## Author contributions

SL: Writing – original draft, Writing – review & editing. XL: Writing – review & editing. NB: Conceptualization, Writing – original draft. DW: Investigation, Writing – review & editing. WY: Conceptualization, Writing – original draft. FW: Writing – review & editing. HJ: Investigation, Writing – original draft.
